# Employment status of patients with schizophrenia spectrum disorder treated with long acting injectable paliperidone palmitate: Real world mirror image study

**DOI:** 10.1192/j.eurpsy.2022.1880

**Published:** 2022-09-01

**Authors:** M. Markovic

**Affiliations:** Clinic for mental disorders ”Dr Laza Lazarevic”, Emergency Department, Beograd, Serbia

**Keywords:** long acting injectable antipsychotic, Paliperidone palmitate, Employment, schizophrenia spectrum

## Abstract

**Introduction:**

Schizophrenia spectrum disorders may severely limit ability to achieve and maintain gainful employment of affected working-age individuals.

**Objectives:**

Assess the employment status in patients with schizophrenia spectrum disorders treated with long acting injectable paliperidone palmitate after the switch from oral antipsychotics.

**Methods:**

A single centre mirror image design study of 115 patients with schizophrenia spectrum disorder was conducted in a tertiary level psychiatric hospital. Data were collected for period of 12 months prior toand 12 months after switching from oral antipsychotic to long acting injectable paliperidone.Employment status for 6 enrolled patients was missing.

**Results:**

Mean age of enrolled patients was 38,4±11,6 years. Of the 109 patients analyzed for employment status, 44,4% remained employed for 12 months after switching to long acting injectable paliperidone while 4,6% patients changed their employment status from unemployed to employed after the switch. No patient changed their employment status from employed to unemployed after the switch. 9,2% patients were already retired at the beginning of study period and 5,5% of patients maintained their student status. 36,7% patients remained unemployed for the whole study period. The correlation between employment status of employed and unemployed patients and duration of illness was borderline significant with p=0,049.

**Conclusions:**

The data from this study suggest that use of long acting injectable paliperidone contributed to preservation of working ability of working-age patients suffering from schizophrenia spectrum disorders.

**Disclosure:**

No significant relationships.

